# Medical student’s experiences of communication with dying patients and their families

**DOI:** 10.1186/s12909-025-08297-y

**Published:** 2025-11-29

**Authors:** Isaac Frost, Hugh Alberti, Rebecca Holdsworth

**Affiliations:** 1 School of Medicine, Newcastle University, Newcastle upon Tyne, England UK; 2 National Institute for Health and Care Research, London, England UK

**Keywords:** End of life, Communication, Education, Medical student, Dying, Palliative care, Undergraduate, Experience, Challenges

## Abstract

**Introduction:**

Effective communication with dying patients is an essential skill for doctors at all stage of their careers. However, many newly qualified doctors remain unprepared to communicate with dying patients and their families. This study aimed to explore medical students’ experiences and challenges in communicating with dying patients and to identify strategies they perceive could improve their communication skills. The motivation for this research stemmed from the personal experience of the lead author: despite an interest in the topic, he had found that he lacked the necessary tools to handle these situations confidently. This study sought to explore whether other medical students share this feeling, and thus adding a novel contribution to the field.

**Methods:**

Medical students from years three to five were invited to participate in semi-structured individual interviews. A total of eight interviews were conducted and used in the final analysis. Interview recordings were transcribed verbatim and reflexive thematic analysis was undertaken following the Braun and Clarke model.

**Results:**

Three key themes were constructed from the data: (1) the importance of communication with dying patients, (2) the challenges faced by students, including inadequate teaching, gaps in communication skills, clinical variability, emotional impact and complexity in communication, and (3) strategies for improving education, with students suggesting course improvements to better prepare them for future clinical practice.

**Discussion:**

Medical students feel unprepared to communicate with dying patients, citing limited exposure, emotional difficulties and insufficient teaching. This study highlights the need for: improved medical training that prioritises communication with dying patients, earlier and increased student exposure to these situations and further research to determine effective strategies to resolve this issue. By addressing these gaps, we can improve the care provided to dying patients and their families, ultimately fostering greater trust between families and healthcare providers.

**Supplementary Information:**

The online version contains supplementary material available at 10.1186/s12909-025-08297-y.

## Introduction

 In the evolving landscape of medicine, the ability to care for dying patients is an essential skill [1, 2]. Yet, despite thirty years since the first version of the GMCs document ‘Tomorrows Doctors’ being released, which outlined palliative care as a core skill for undergraduate training [2, 3], many newly qualified doctors remain unprepared to communicate with dying patients and their families [4–7]. Medical students have difficulties and discomfort in talking about dying with patients and their family members [8]. These students will soon face caring for on average forty dying patients and an additional 120 end-of-life care patients within their first year of work [9]. This gap in training is not just a flaw; it is a serious issue that directly impacts on quality of patient care.

The UK department of health report ‘*More Care*,* Less Pathway: A Review of the Liverpool Care Pathway*’ proposed to improve communication with dying patients and their families by doctors [10]. This report highlighted poor communication as a recurring issue, in some cases leading to distress and unnecessary misunderstandings in the last days of a patient’s life [10]. Poor communication can lead to suboptimal care, subjecting patients and their families to undue mental and physical anguish, whereas good communication can ease pain and suffering whilst giving patients the chance for a more peaceful death [10]. Open and direct discussions with dying patients and their families not only alleviate fears but also strengthen family relationships and reduce the isolation often experienced by the dying person [10]. By enhancing junior doctor’s ability to have conversations with those near death, positive connections with patients can be forged and developed, making their roles more rewarding whilst also minimising the patient’s pain and suffering [10].

Current medical education lacks training in communicating with dying patients and provides insufficient emotional support for students [11].This gap in education may contribute to the compassion fatigue and burnout seem amongst some medical professionals [12]. Improving the curriculum to include comprehensive training and support for communication with dying patients and their families is reported to enhance patient outcomes and reduce burnout [10, 13].

There is robust literature suggesting that medical students’ experiences of professional loss can contribute to their development as physicians [14, 15]. This learning often occurs through witnessing death during clinical placements and processing these emotionally significant events independently. Students emphasised the importance of empathy and compassion, developing a deeper appreciation of the impact of death and dying on patients and their families, and described acquiring practical skills such as improved communication and the ability to manage personal emotions [14, 15].

Studies have not adequately explored how medical students learn about end-of-life care or how newly qualified doctors develop the skills necessary to communicate with dying patients and their families [1]. Existing teaching is decentralised and often unassessed, leading to a lack of consistency on what is taught about end-of-life care between medical schools [2]. The processes of learning and the most effective teaching methods remain unclear due to a lack of evidence from the literature [2]. Supervising doctors often protect students from exposure to dying patients or feel uncomfortable delivering training in this area [1]. As a consequence, medical students frequently lack sufficient exposure to dying patients, a challenge common to all medical schools, regardless of the level of end-of-life care taught outside of placement [1]. On graduation, medical students express concern about their ability to care for dying patients and report low satisfaction with both the quality and quantity of end-of-life education [11]. This is despite generally favourable attitudes towards palliative care, highlighting the main shortcoming in current teaching: insufficient academic and clinical exposure [16]. A key factor contributing to this is their limited exposure to dying patients in the clinical environments, leaving them with insufficient practical experience [17]. As a result, they are often not read to provide end-of-life care, which can cause unacceptable grief and hardship for patients and their families [4, 10, 12]. Given the numerous challenges faced, there is a clear need for changes in undergraduate medical education to better prepare future doctors for more effective end-of-life care [11].

### Aims

This study aims to explore medical students’ perspectives on their undergraduate education regarding communication with dying patients and their families. By gaining insights into students’ experiences, teaching methods and communication strategies, we can identify areas for improvement. Enhanced guidance and training in communication with dying patients and their families may allow more peaceful end-of-lives for patients and their families and potentially alleviate pain and suffering [10].

## Methodology

### Theoretical framework

Communicating with a dying patient and their family is an inherently subjective task, influenced by personal history and individual experiences [18]. Participants’ responses and perceptions are shaped by unconscious memories and past interactions [19], aligning this study with a constructivist approach, which recognises that knowledge is actively constructed through lived experience [20]. Accordingly, this research adopts a subjective epistemological stance, acknowledging that knowledge is context dependent and shaped by each participant’s unique perspective [21]. A relativist ontological paradigm underpins this stance, assuming multiple social realities exist, each shaped by culture, experience and individual interpretations and are open to change over time [21].

These theoretical positions directly guided the research design and methodology. Semi-structed interviews were chosen to allow participants to narrate their experiences in their own terms, enabling deep exploration of individual meaning-making. The use of reflexive thematic analysis aligned with constructivist orientation, acknowledging the co-construction of meaning between researcher and participant. Reflexivity was central to this process, with attention paid to the researchers positionality and its influence on data interpretation.

By integrating these positions, the study aimed to understand how medical education can be improved to enhance end-of-life communication whilst exploring individual meaning and how experiences influence participants’ responses.

### Research design

This study employs an inductive, exploratory qualitative research design using semi-structured interviews to explore medical student’s views on developing communication skills with dying patients and their families [22]. Semi-structured interviews enable the researcher to deeply explore personal matters while also allowing flexibility to deviate from standardised questions. This approach helps elicit key information tailored to the interviewee’s unique knowledge and experience [23]. This study is cross-sectional, capturing participants’ experiences and perspectives at a single point in time. An inductive approach allows for themes to be constructed from the data without imposing pre-existing theories or frameworks [22].

### Participants

Participants were chosen using purposive sampling, by selecting volunteers who have relevant stories to share, enabling the selection of information rich cases that highlight experiences of central importance to the study [24]. Inclusion criteria was clinical year (3rd, 4th and 5th) medical students with experiences of communicating with dying patients and their families. Recruitment was conducted via email invitations from the central medical school, followed by verbal invitations.

Saturation in qualitative research is a debated concept, particularly regarding when and how it is reached. This study aligned with the concept of data saturation – the point at which no new codes or insights emerge from additional data. While full saturation may never be completely attainable, and some degree of uncertainty is inevitable, we observed that very few new codes were generated from the seventh and eighth interviews. We therefore judged this point to represent a satisfactory level of data saturation for the purposes of this study [25].

### Data collection

An interview guide (Supplementary File 1) was developed and used for this study only, based on a literature review and expert consultation, including open-ended questions such as “How confident would you be communicating to a dying patient?” [26]. Interviews were held over Microsoft Teams lasting between 25 and 50 min [27]. All interviews where audio-recorded and transcribed verbatim.

### Data analysis

Data was analysed using reflexive thematic analysis following Braun and Clarke’s framework [28]. This method was selected to draw a clear, consistent narrative from the data about communicating with dying patients, with the aim of drawing conclusions on how to better educate future medical students [29].

The inductive approach chosen involved generating themes directly from the data. Transcripts were read multiple times to allow for deep familiarisation. Initial coding was performed line-by-line to identify significant statements and patterns. Some examples of initial codes included *“lack of experience*,*” “inadequate teaching*,*” “importance of empathy*,*”* and *“avoidance of discussing death.”* These codes were then constructed into potential themes.

To enhance the trustworthiness of the analysis, several strategies were employed. Investigator triangulation was supported through regular discussions with co-authors (HA and RH), who reviewed coded data and contributed to the development and refinement of themes, helping to reduce individual bias. Data triangulation was achieved through collaborative data analysis and comparison of participant perspectives across interviews to ensure consistency. A reflexive approach was maintained throughout, with attention given to how the first authors positionality as a medical student may have shaped data interpretation. Themes were regularly checked against the raw data to ensure alignment with participants accounts, strengthening dependability and confirmability [30]. NVivo software facilitated data organisation and analysis [31].

### Reflexivity statement

The first author (IF) entered this project with the assumption that training on communicating with dying patients and their families in medical school was insufficient, based on personal experiences and special interest in the topic. This positionality may have influenced design of interview questions, interpretation of response and prioritisation of themes during data analysis [32].

To ensure reliability and validity, IF revisited the raw data and engaged in ongoing critical discussions with co-authors - HA (an experienced educationalist researcher) and RH (an early-career educationalist with a particular interest in death and dying). This collaborative process helped maintain reflexive awareness and supported a commitment to preserving participants’ intended meaning [33]. Both HA and RH have prior clinical experience providing end-of-life care. This experience may have influenced the revision of themes and interpretation of findings during the discussion, as their past experiences could shape how they perceive and analyse the data.

As a fellow medical student, IF shared a training environment and cultural context with the participants. This insider status may have helped reduce power imbalances, facilitating more open and honest exchanges [32]. Additionally, the voluntary nature of participation and the use of online interviews may have further minimised any perceived coercion or hierarchy.

HA and RH supported IF’s initial concerns and reasons for entering the research and engaged in regular discussions with IF throughout the process to help explore and manage emerging reflexive concerns.

### Ethical consideration

This study received approval through Newcastle University Faculty of Medical Sciences Research Ethics Committee (Ethics application 2492/29356), in conjunction with a larger research project on dying experiences and learning. Informed consent was obtained from all participants, ensuring confidentiality and the right to withdraw at any time. Identifiable information was anonymised, and data was securely stored. Given the sensitive topic, participants were informed they could skip questions or stop the interview at any time, with support resources (Appendix 3) provided for potential distress.

## Results

Eight interviews were undertaken with students from years 3,4 and 5. Interviews lasted between 25 and 50 min and participants details are listed on Table 1, which presents the demographic characteristics of the sample. The eight participants were all medical students from Newcastle University. Half of the participants (50%) had recently completed their fourth year of medical education, with 25% each had recently completed years 3 and 5. The sample included five females and three males, with ages ranging from 22 to 34 years of age. There was an even distribution between undergraduate and graduate entry medical students, allowing for a range of perspectives based on educational background and stage of training.

**Table 1:** Demographics of participants

Participant	Age	Stage of Medical School	Gender	Medical school programme
P1	26	Year 3	Male	Graduate entry
P2	30	Year 4	Male	Graduate entry
P3	27	Year 5	Female	Graduate entry
P4	22	Year 4	Male	Undergraduate
P5	34	Year 4	Female	Undergraduate
P6	28	Year 5	Female	Graduate entry
P7	22	Year 3	Female	Undergraduate
P8	22	Year 4	Female	Undergraduate

**Fig. 1:**
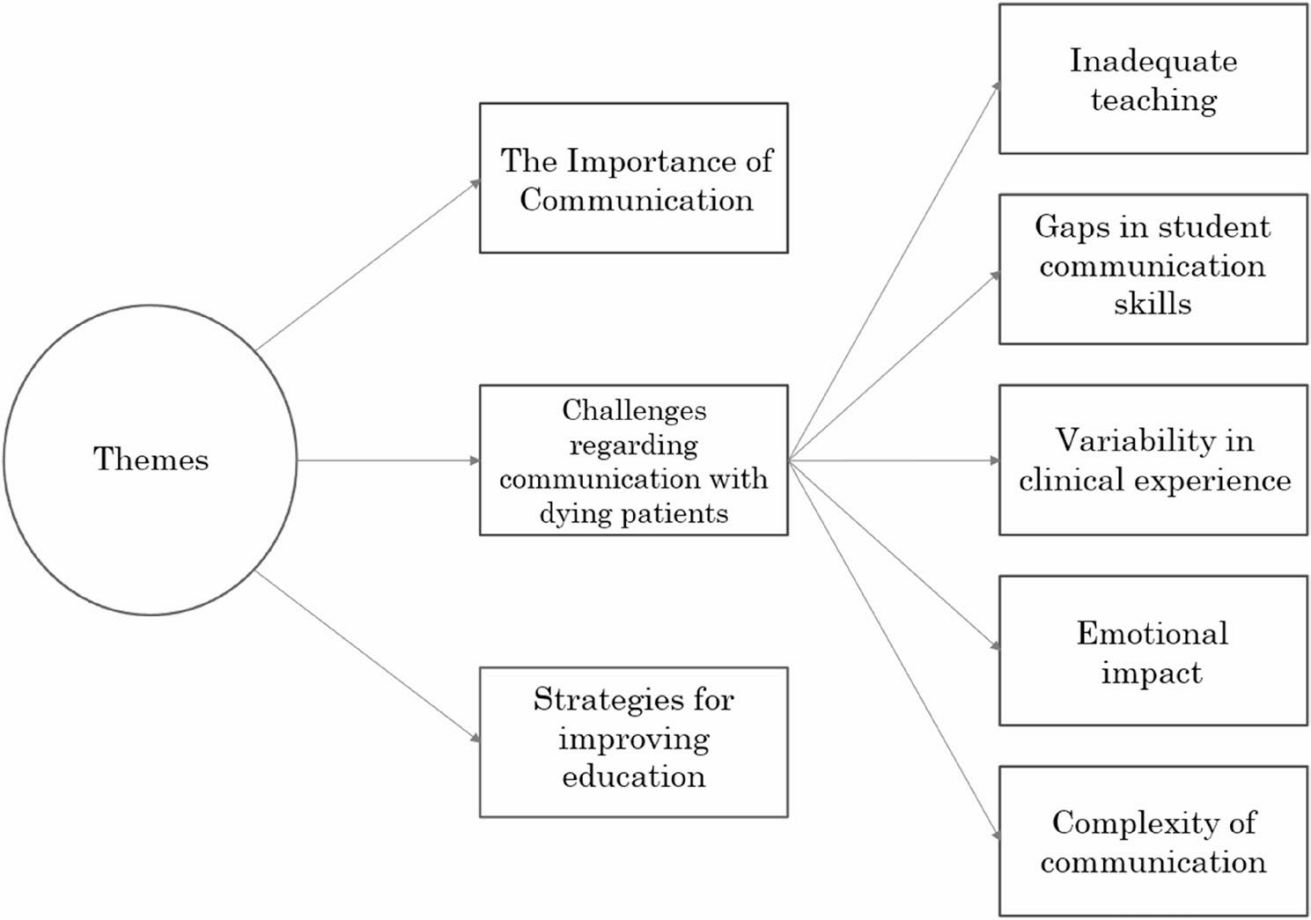
Research themes

Figure 1 provides a visual summary of the key themes generated from reflexive thematic analysis. Three main themes were constructed, with Theme 2 containing five subthemes, which are represented as branches extending from Theme 2 in this figure. This thematic structure offers a framework for understanding the participants responses related to communication with dying patients and their families.

### Theme 1: importance of communication

While medicine is a broad field with varying priorities, communication with dying patients and their families is acknowledged as a *“big factor”* amongst participants.

*“You’re going to be dealing with it all the time”. (P8)*

*“Generally*,* whatever ward you walk onto*,* there’s probably always a patient on end of life on the ward at any one time.” (P5)*

The students felt that the current lack of emphasis on teaching these skills is disproportionate to the frequency and significance of their application in both undergraduate education and professional practice.

*“It can really affect the rest of the interactions and rapport with the patient”. (P3)*

*“If you don’t know how to navigate death*,* you’re going to struggle” (P2)*

When asked about communication, they stressed the fact that the doctor-patient relationship is fundamentally built on communication. The impact should not be understated.

*“It doesn’t matter if you’re derm*,* it doesn’t matter if you’re ophthalmology you know … your interest doesn’t even matter in this case because you’ve got to deal with someone who’s dying. You’re going to deal with someone who’s dead and have to speak to their family*,* right?” (P4)*

The understanding of participants was that every doctor has the responsibility to be able to communicate effectively with the dying patient and their family no matter the speciality.

*“It’s also a big responsibility.” (P2)*

Participants discussed the privilege it is to be able to care for end-of-life patients.

*“It’s quite a privilege to be able to care for people when they are vulnerable.” (P6)*

*“You’re in a privileged position. You’re here to help people.” (P2)*

### Theme 2: challenges regarding communication with dying patients

Participants discussed the challenges they faced in their experiences when communicating with dying patients and their; five subthemes were constructed:

### Sub-theme 1: inadequate teaching

There was a consensus that the university’s teaching provided insufficient education on communication with dying patients. The lack of focused communication training, *“vague”* mark schemes and avoidance of death-related discussions in clinical settings contributed to students being unprepared.

*“I think the teaching they give on any kind of communication skills is lacking” (P4)*

*“There is no communication on how to actually have that end-of-life conversation” (P5)*

*“Even outside of that one teaching session*,* I never got any teaching from other stuff” (P1)*

*“We’ve not had any formal training at all*,* I’d say” (P7)*

*“you don’t really go through a lot of teaching on how to deal with it yourself or even exactly what’s going to happen” (P3)*

*“Would you say that medical education hasn’t prepared you at all for these conversations?” (Interviewer)*

*“100%” (P2)*

It was expressed that the lack of teaching may be due to the potential for these conversations to be uncomfortable.

*“They skirt over it really quickly because it’s uncomfortable*,*” (P5)*

### Sub-theme 2: gaps in student communication skills

The deficiency in teaching and hands-on experience resulted in significant gaps in students’ communication abilities with dying patients. This created a snowball effect: students who lacked these skills tended to avoid difficult conversations, feel confused about their roles and struggle to initiate them.

*“I was like a rabbit in headlights”*,* (P5)*

*“very confusing time … I had no idea what was going on”. (P4)*

*“I don’t know what question I should ask them …. How do you communicate with them?” (P1)*

*“I know that a lot of people* (medical student colleagues) *don’t really know what to say to a dying patient” (P5)*

The impact of the lack of guidance from educators is evident; participant seven spoke about being *“horrified”* to lead conversations and participant two acknowledged,

*“I think we shy away from death”. (P2)*

### Sub-theme 3: variability in clinical experience

*“Exposure and experience on the ward is so very*,* very important”*,* (P5)*

This theme details the high variability of clinical exposure in medical school. This is, in some capacity, due to the limited time dedicated to observing doctors having conversations with dying patients. However, the chance to gain this experience is often dependent on placements and mentors.

*“I know I have a lot of friends* (medical students) *who’ve never seen someone die at my stage” (P4)*

Participant three mentioned that it’s *“purely by chance”* that students encountered mentors who allowed them to gain such experience. This can particularly impact younger students who may lack life experience, reinforcing the need for more exposure to help them grow.

*“Do you feel prepared to communicate with dying patients and their families?” (Interviewer)*

*“No … especially for the younger students are coming straight through university*,* probably don’t have that experience” (P5)*

Variability in teaching between base units, where you are placed for the year, was also noted by participants.

*“Unfortunately*,* in my LEP*,* my learning education provider*,* we don’t have any formal teaching in palliative care and conversation with dying patients.” (P1)*

### Sub-theme 4: emotional impact

Dealing with the emotional impact of witnessing and participating in a patient’s death is another significant challenge for the participants. For many, this may be the first death they have encountered, or it may trigger memories of a loved one’s passing.

*“You feel out of your depth*,* you feel vulnerable*,* you feel helpless” (P2)*

*“I literally had to go off to the side so the mum wouldn’t see me tear up because I saw her like cry her eyes out” (P4)*

*“Certain cases hit you a little harder*,* and this one hit me quite hard that night*,* to the point where I even cried myself” (P5)*

*“I didn’t process it*,* but afterwards I was like really upset” (P8)*

The gravity of these emotions stopped students from learning these skills. Despite this challenge, students reported that working with dying patients, when it occurred, was an *“honour”* and a *“privilege”.*

### Sub-theme 5: complexity in communication

The final theme shows understanding that communication, particularly around death, is complex.

*“Something like death*,* it doesn’t fit into a nice*,* neat box” (P2)*

Each situation is unique, influenced by the social dynamics of the patient, their illness and the healthcare team.

Time constraints doctors face can make it difficult to engage in deep, meaningful conversations with dying patients. *“It’s just not possible”*, participant five remarked, referring to the limited time available spent with patients. Moreover, doctors often have little time to reflect on challenging cases, forcing them to *“compartmentalise”* their feelings and quickly move onto the *“next patient and the next patient”.*

*“They’ve got many*,* many different patients to see they can’t really be spending most of the time with one grieving family when they might have five patients like this elsewhere scattered around the hospital” (P5)*

Communication with dying patients is further complicated by involvement of their families, who may not have been included in the conversation early enough. Participant six said,” *the family don’t realise how like sick the patient is*”. This can lead to frustration and even “*aggression*” from family members and may cause students to feel fear and discomfort in these situations.

*“I try to avoid directly speaking to their family*,* that’s what I do*,* because I’m still not confident on how to speak to them.” (P1)*

### Theme 3: strategies for improving education

The final theme is based upon proposals by students for improving education, presented here by significance among participants.

Training from health care professionals with expertise in end-of-life care was the most frequently requested strategy. Two interviewees spoke highly of the impact of a specific workshop in comparison to their curriculum.

*“That event is ten times better than anything that the uni’s done to prepare us for this.” (P8)*

*“Palliative nurses are very*,* very good at teaching” (P3)*

Specialists were regarded as the “*best people*” to teach communication with dying patients and their families.

In addition to training in non-clinical settings, there was a strong desire among students for more palliative care placements. They felt that real-world experience was essential for their development. Participant six noted that students should not “*shy away from it*.” They went on to say,

“*if there’s an opportunity to observe anybody going and having these conversations*,* we should all try and do that.” (P6)*

*“I think exposure and experience on the ward is so very*,* very important” (P5)*

*“Because I had that experience*,* I kind of got a bit more used to the end of life and palliative side of it” (P3)*

Roleplay and simulation were also highlighted as effective strategies, enabling medical students to practice their skills in a safe environment.

*“You have to practice on someone*,* that’s the big thing”. (P4)*

*“With actors*,* I think is really useful because it gives you the kind of safety to practice” (P7)*

These methods were seen as valuable means of letting students gain exposure without the risk of upsetting patients with mistakes.

Cultural awareness was felt as another significant topic. Due to the diversity in backgrounds of patients, understanding the various cultural and religious perspectives on death was perceived to be an increasingly important part of a doctor’s role. This requires a need for education that promotes equitable care for all dying patients.

*“The understanding about how to navigate those cultural sensitivities is something I think would help” (P2)*

*“Social and cultural awareness would then help them tailor their consultation to helping the family of the deceased and the person dying” (P2)*

## Discussion

Our study, the first UK based qualitive study into medical students’ experiences of communication with dying patients and their families, has revealed key themes regarding medical students’ perspectives on communicating with dying patients.

Effective communication in these contexts is perceived to be an essential skill; however, it remains underrepresented in our medical school’s curriculum. Students often feel unprepared and unsupported mainly due to challenges within medical education. Alarmingly, many of our students go through medical school without any direct experience of talking to dying patients. Students tend to avoid engaging with dying patients whenever possible, further indicating their lack of experience and confidence in managing end-of-life situations. This avoidance reflects both their limited exposure to, and discomfort with interacting with dying patients [1, 6, 34, 35]. Without structured guidance, our graduating students enter their first year as junior doctors (Foundation year one in UK) inadequately prepared, which can lead to suboptimal care.

Various challenges were noted by participants, each characterised by its own unique complexities. The emotional impact of dealing with death was a common struggle amongst participants. A systematic review has previously described junior doctors reporting intense experiences with death, which can lead to feelings of depression, anxiety and dread [11]. Medical schools providing early, supervised exposure could potentially help students adapt as they begin their careers. A study examining graduating students’ knowledge and perspectives regarding personal experiences with death found that those who had been exposed to such experiences previously exhibited more positive attitudes and greater knowledge compared to those who had not [36]. A potential solution therefore would be a greater focus on prioritising emotional preparedness, as it can significantly influence students’ professional development and their ability to provide compassionate care.

Our participating students’ spoke of variability in their clinical experiences which stemmed from the individual placements and mentors assigned to each student. While other studies have spoken of the differences between teaching at individual medical schools, the impact of mentor variability and placements throughout the five years of medical school is less commonly spoken of in the literature [1, 37]. Placements vary in both the number of patients receiving end-of-life care and the degree of exposure facilitated by mentors, which affects how much students can observe or participate in patient care. This variability presents a significant challenge as some students may therefore get no or very little exposure of death in their time at medical school.

A previous report has identified lack of time as a significant barrier to health-work conversations for doctors [38]. The findings of this study align with this view, emphasising that students perceive that doctors often struggle to engage in deep, meaningful conversations with dying patients due to time constraints. This limitation is likely to affect doctors’ ability to communicate effectively with patients, despite any improvements in education at a medical student level. Doctors have concerns about having time for sensitive discussions and studies show time constraints have been found to compromise effective end-of-life patient care [39, 40].

Participants emphasised the lack of formal teaching, the result of which has caused participants to tend to avoid these conversations due to a perceived lack of knowledge. As one systematic review put it, “junior doctors are thrown in at the deep end when it comes to end-of-life care” [11, 41]. Consequently, they must learn on the job, often struggling to effectively communicate with dying patients during their initial experiences, which may result in negative outcomes for both the patients and the healthcare providers involved.

Participants discussed the complexity of communication in end-of-life situations, recognising that each interaction with a dying patient is unique. As a result, they emphasised that the teaching cannot follow a one-size-fits-all approach. This observation further stresses the importance of direct exposure to dying patients, as opposed to relying solely on conventional teaching methods such as seminars or lectures. Most participants spoke of the value and importance of acquiring communication skills specific to end-of-life care – a finding consistent with existing literature, which suggests that medical students recognise the importance of effective communication in these settings [42]. Participants also proposed several strategies for enhancing education in this area, offering a foundation for future research aimed at identifying the most effective ways to prepare students for these conversations.

A further novel finding of this study was the impact families had on medical students’ experiences. Some participants expressed a tendency to avoid speaking with patients’ families due to the perception that families may be aggressive and confused due to being unaware of how sick their family member is. We found that the perceived fear of patients’ family’s reactions can be a barrier to students wanting to gain experience with talking to dying patients – a unique finding of this research. This aspect has rarely been covered in the literature [43]. especially in regard to medical students, indicating a need for further research.

### Strength and limitations

This research provides new insights into the experiences of medical students that can inform improvements to our existing curriculum and encourage other schools to review their own curricula. The interviewer’s status as a student helped create a comfortable environment for participants to express their feelings without the influence of power imbalances. Although the lead author was a relatively novice researcher he was supported by two experienced colleagues.

We acknowledge that the data was collected from students at a single medical school, limiting its findings to one region and curriculum. We encourage similar studies in other institutions to enhance the robustness of this research.

Some perspectives from the university may not be represented in this study. Since participation was voluntary, it is possible that students who had particularly positive, challenging or more neutral experiences, chose not to take part. Additionally, those who did volunteer may have been individuals with special interests or strong opinions and therefore not representative of the wider medical cohort.

Another limitation of this study is the variation in participants stage of medical education. Students from years 3,4 and 5 have differing levels of clinical exposure, which may influence their experiences and communication skills in relation to end-of-life care. These differences could have shaped the depth and focus of their responses, as exposure to certain clinical settings and situations often occurs in the later years of training. This variation may affect the comparability of some responses across participants.

### Implications

Medical students need increased exposure and experience in communicating with dying patients before they begin their professional practice. Medical schools should explore ways to prioritise this aspect of the curriculum, enabling students to engage more frequently with end-of-life communication. Participants in this study consistently emphasised that such exposure would enhance their communication skills. Evidence also suggests that additional conversations about values, goals and prognosis – when introduced earlier in the treatment journey - can alleviate symptoms of anxiety and depression among carers, which in this context includes medical students [44]. By integrating more meaningful teaching alongside increased exposure, medical students are likely to overcome their tendency to avoid these conversations and feel that their contributions are valuable to patients as well as building up a portfolio of experience to draw upon in future practice.

Educators, including mentors, clinicians and teaching fellows, should take a more proactive approach in facilitating student interactions with dying patients. By involving students in these conversations, we can enhance the communication skills of future clinicians, ultimately improving the quality of care for future patients. Moreover, patients may benefit from engaging with students who can dedicate more time to meaningful discussion at the end-of-life.

While there is substantial literature on educating medical students in communication with dying patients, further research is desirable to evaluate the real-world impact of this training. Further research authored by medical students is desirable, as their embedded position within the learning environment allows them to rapidly identify challenges and propose practical solutions. Exploring the outcomes for patients resulting from these educational initiatives will support a greater investment of time in the education and curriculum surrounding end-of-life care.

## Conclusion

Our study provides a novel insight into the experiences of current medical students around end-of-life and care for dying patients’ communication. To conclude, the reality is that senior medical students, despite being close to entering their professional roles, appear to lack the experience to discuss death with patients and their families, a challenge that threatens the quality of future care. Without meaningful change, newly qualified doctors will continue to avoid conversations, be uncertain about their role and ultimately, and patients and their families will potentially. Medical education must prioritise increased early exposure for students and increase the quality and quantity of students experience throughout the rest of their training.

## Supplementary Information

Supplementary Material 1.

Supplementary Material 2.

Supplementary Material 3.

Supplementary Material 4.

## Data Availability

The datasets generated and/or analysed during the current study are not publicly available to ethical and confidentiality considerations. Participants did not provide consent for their identifiable information to be shared outside the research team, and the study’s ethical approval does not permit the release of this data.
