# Supplementary material for: Medical student’s experiences of communication with dying patients and their families
Source: BMC Med Educ. 2025 Nov 29;26:3. doi: 10.1186/s12909-025-08297-y (PMC12771949; doi:10.1186/s12909-025-08297-y)
Supplement: Supplementary file 1 — Supplementary Material 1. [file 12909_2025_8297_MOESM1_ESM.docx]

Interview Guide – Exploring Medical Students Views on Communication with dying patients and their families

Introduction

Demographic questions –

Age?

Mature student?

Gender?

Stage of medical education?

Opening questions

1. Why did you choose medicine as a career?
2. What field of medicine do you want to work in? Why?
3. How much experience have you had with patients in end-of-life situations?

Experiences with dying patients and families

1. Have you had any specific experience with a dying patient? Can you explain how you felt during that time? Have your feelings changed over time? Why?
2. What challenges were faced in this encounter, especially in terms of communication? Why?
3. How did these challenges impact the ability to communicate with the patient and their family? Why?

Communication

1. Do you feel prepared to communicate with dying patients and their families?
2. How confident would you be to communicate with a dying patient?
3. What aspect would you feel most confident in?
4. What aspect would you find most challenging? Why?

Strategies

1. Have you had any teaching on communication with dying patients? What was it?
2. How has this influenced your encounters? Why?
3. How have you learned to communicate with dying patients? What did you take away from these experiences?
4. Why was this learning significant?
5. In what way has your medical education prepared you for these conversations?

Improvements

1. Based on your experiences, what suggestions do you have for improving end-of-life communication training in medical education? Why?
2. How important do you think communication training with the dying patient would be?
3. Are there any specific parts of end-of-life communication that you believe should receive more focus in medical education? Why?
4. What would be the best way to provide any new training? Why?
5. What could have been done to better prepare you for end-of-life communication? Why?

Closing questions

1. What advice would you give to other medical students who are about to encounter their first experience with a dying patient?
2. Is there anything else you would like to share?
3. Do you have any final questions or comments?
4. Any feedback?
