# Supplementary material for: Medical student’s experiences of communication with dying patients and their families
Source: BMC Med Educ. 2025 Nov 29;26:3. doi: 10.1186/s12909-025-08297-y (PMC12771949; doi:10.1186/s12909-025-08297-y)
Supplement: Supplementary file 2 — Supplementary Material 2. [file 12909_2025_8297_MOESM2_ESM.docx]

*
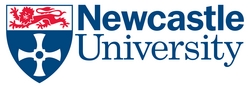
*Participant information sheet:

**Exploring medical students views on communication skills with dying patient and their families**

You are being invited to take part in a research project being conducted by the School of Medicine at Newcastle University.

**Purpose of the research**

The aim of this research is to explore medical students experiences of communicating with dying patients and their families. It will explore personal and professional reflections and encounters during medical training.

**Who is funding the project?**

Newcastle University are funding the project.

**What is involved in participating?**

You are being invited to take part in an interview to discuss your experiences of communicating with dying patients and their families. The interview will take around twenty to thirty minutes.

**Benefits and risks**

Your participation will help us hear the views from a range of medical students to gain more understanding about your experiences. There is a risk that talking about dying and death could be distressing. If you have any concerns, you can discuss these with the lead researcher who can signpost you to useful resources. Participants data will be treated confidentially, except, in the very unlikely circumstance where all researchers agree there is a significant concern about patient safety and/or a breach of GMC duties of a doctor.

**What will happen to my data?**

The questionnaire data will be anonymous. The interview, with your consent, will be audio recorded then transcribed. Any individual identifiers will be removed from the transcript to ensure anonymity. The anonymous data will be archived securely on Newcastle University systems where only named researchers can access it. With your permission, the data will be kept for further research.

# Terms for withdrawal

You may withdraw from the study by leaving the interview at any time, without explanation. Withdrawing will not penalise you in any way.

# Who has reviewed and approved the project?

The study proposal and documentation has been granted approval via Dr Rebecca Holdsworth’s work.

# Staff Researchers’ contact details

If you would like to know more about the research study, including when any findings may become available, please contact: *Redacted.*
