# Supplementary material for: Medical student’s experiences of communication with dying patients and their families
Source: BMC Med Educ. 2025 Nov 29;26:3. doi: 10.1186/s12909-025-08297-y (PMC12771949; doi:10.1186/s12909-025-08297-y)
Supplement: Supplementary file 3 — Supplementary Material 3. [file 12909_2025_8297_MOESM3_ESM.docx]

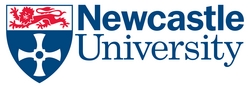
Consent form for interview:

**Medical students views on communication with dying patients and their families**

**This interview is to explore medical students experiences of communicating with dying patients and their families. The researchers require your consent for the interview to be recorded and analysed, and for anonymous quotes to be included in potential future presentations and publications.**

**Please read the points below and if you are happy to provide this consent, sign at the bottom.**

I, the undersigned, confirm that (please initial box as appropriate):

| 1. | I have read and understood the information about the project, as provided in the Information Sheet dated July 2024. |  |
| --- | --- | --- |
| 2. | I have been given the opportunity to ask questions about the project and what my participation involves. |  |
| 3. | I voluntarily agree to participate in the project. |  |
| 4. | I understand I can withdraw at any time without giving reasons and that I will not be penalised for withdrawing. |  |
| 5. | I agree to the recording and transcription of the interview. |  |
| 7. | The procedures regarding confidentiality have been clearly explained to me (i.e. that all data will be anonymised). |  |
| 8. | The use of the data in research, publications, sharing and archiving has been explained to me. |  |
| 9. | I understand that other researchers will have access to this data only if they agree to preserve the confidentiality of the data and if they agree to the terms I have specified in this form. |  |
| 10. | I, along with the Researcher, agree to sign and date this informed consent form. |  |

**Participant:**

___________________ _________________________ ________________

Name of Participant Signature Date

**Researcher:**

_________________ _________________________ ________________

Name of Researcher Signature Date
