# Supplementary material for: Medical student’s experiences of communication with dying patients and their families
Source: BMC Med Educ. 2025 Nov 29;26:3. doi: 10.1186/s12909-025-08297-y (PMC12771949; doi:10.1186/s12909-025-08297-y)
Supplement: Supplementary file 4 — Supplementary Material 4. [file 12909_2025_8297_MOESM4_ESM.docx]

*
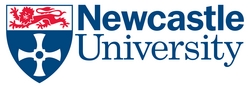
*Support resources

**Medical student views on communication with dying patients and their families**

Thank you for taking part in this study. If you have found reflecting and discussing your experiences distressing in any way, we would encourage you to seek further support.

If you require urgent support, we recommend visiting your local Emergency Department or contacting your GP.

**Medical Student Office**

The Medical Student Office is open to help any medical students with their needs. They can signpost to more resources or get you in touch with the relevant persons needed. Email *redacted* or call *redacted* (5 days a week, 9am-5pm)

**Student Wellbeing**

A free, confidential service for students of Newcastle University with any wellbeing problems and plenty of services. *Redacted*

**Papyrus Hopeline247**

This support line runs 24 hours a day, seven days a week to act as a crisis line for anyone wishing to talk to professionals about any thoughts of suicide or poor mental health. Lines are open every day of the year on 0800 068 4141.

**Bereavement support, operated by Hospice UK**

A confidential, free, bereavement support line from **8am – 8pm, seven days a week on 0300 303 4434. This is run by a** fully qualified and trained bereavement specialists to support you with bereavement and wellbeing issues relating to loss experienced through your work.

**Mind**

A registered charity which offers advice and support to anyone experiencing a mental health problem. Call on 0300 123 3393 or email info@mind.org.uk or visit their website www.mind.org.uk

**Tea and Empathy Facebook Group**

A supportive, active network of healthcare professionals who offer real life, non-judgemental advice on a variety of topics which can be anonymous.

Search ‘Tea & Empathy’ in Facebook.

**Further information on the study**

Thank you again for taking part. If there is anything you would like to discuss in relation to this study, please feel free to do so by contacting the researchers. If you would like to withdraw your data, please email the lead researcher *Redacted*.
